# Identifying the Candidates Who Will Benefit From Extended Pelvic Lymph Node Dissection at Radical Prostatectomy Among Patients With Prostate Cancer

**DOI:** 10.3389/fonc.2021.790183

**Published:** 2022-01-26

**Authors:** Guanjie Yang, Jun Xie, Yadong Guo, Jing Yuan, Ruiliang Wang, Changcheng Guo, Bo Peng, Xudong Yao, Bin Yang

**Affiliations:** ^1^ Department of Urology, Shanghai Tenth People’s Hospital, Tongji University School of Medicine, Shanghai, China; ^2^ Shanghai Clinical College, Anhui Medical University, Shanghai, China

**Keywords:** lymph node dissection, prostate cancer, prostatectomy, nomogram, survival

## Abstract

**Purpose:**

The therapeutic effect of extended pelvic lymph node dissection (PLND) in prostate cancer (PCa) patients is still controversial. The aim of this study was to identify the PCa patients who may benefit from extended PLND based on the 2012 Briganti nomogram.

**Materials and Methods:**

PCa patients who underwent radical prostatectomy (RP) plus PLND between 2010 and 2015 were identified from the Surveillance, Epidemiology, and End Results (SEER) database. The probability of lymph node invasion (LNI), determined using the 2012 Briganti nomogram, was used to stratify the patients. The endpoints were overall survival (OS) and cancer-specific survival (CSS). Propensity score matching (PSM) was performed to account for potential differences between patients with and without extended PLND. Univariable and multivariable Cox regression was used to analyze the association between the number of removed nodes (NRN) and survival. Kaplan–Meier analysis was performed to estimate OS and CSS. Extended PLND was defined as NRN >75th percentile.

**Results:**

A total of 27,690 patients were included in the study. NRN was not an independent predictor of OS (p = 0.564). However, in patients with probability of LNI ≥37, multivariable analyses showed that increased NRN was associated with improved OS (hazard ratio [HR] = 0.963; p = 0.002). The 5-y OS rate was significantly higher for patients with NRN ≥12 than those with NRN <12 (94.9% vs. 91.9%, respectively; p = 0.015). In the PSM cohort, among patients with probability of LNI ≥37, multivariable analyses showed that increased NRN was associated with improved OS (HR = 0.961; p = 0.004). In addition, the 5-y OS rate was significantly higher for patients with NRN ≥12 than those with NRN <12 (94.9% vs. 89.8%, respectively; p = 0.002). However, NRN was not an independent predictor of CSS in any LNI risk subgroup (all p >0.05).

**Conclusion:**

Extensive PLND might be associated with improved survival in PCa patients with a high risk of LNI, which supports the use of extended PLND in highly selected PCa patients. The results need to be validated in prospective studies with long-term follow-up.

## Introduction

Current imaging techniques have low sensitivity for detecting lymph node metastasis in prostate cancer (PCa) patients. Therefore, pelvic lymph node dissection (PLND) is considered the best method to stage the disease and determine the ideal treatment (1, 2). Although previous studies have demonstrated that the lymph node invasion (LNI) rate increases with the extent of PLND, the therapeutic effect of extended PLND is controversial (3–5).

A number of studies and reviews have reported that extended PLND does not improve the outcomes and leads to a higher risk of complications (6–9). Results from several recent randomized controlled trials (RCTs) suggested that extended PLND did not significantly decrease the risk of biochemical recurrence (BCR), cancer-specific mortality (CSM), and metastasis compared to limited PLND (10–12). There may be two main reasons for the unfavorable results of extended PLND during radical prostatectomy (RP). First, the dissection templates varied between studies. Second, a majority of patients were pathologically node-negative after surgery and at a low risk of cancer progression. In contrast, a few studies showed a survival benefit of extended PLND in some patient subgroups: pN1, high Gleason score (International Society of Urological Pathology [ISUP] grade groups 3–5), intermediate- and high-risk groups, and pT3-T4 PCa patients (10, 13–15). Therefore, the therapeutic benefit associated with extended PLND should be tested in subgroups of PCa patients. Novel prediction models, imaging techniques, molecular classification, and artificial intelligence have high accuracy in detecting lymph node metastasis and may identify patients suitable for extended PLND (2, 16–18). A recent study reported a Node Reporting and Data System 1.0 (Node-RADS) for standardized reporting of possible distant lymph node metastasis on CT and MR imaging (19). The novel system will lead to an increase in the consensus in radiological assessment of lymph nodes in PCa patients and may be used to select suitable patients for extended PLND. The guidelines of the European Association of Urology recommend extended PLND for PCa patients with a risk of LNI exceeding 5% based on the Briganti nomogram (20–22). However, several studies have suggested that the LNI cutoff of 5% may be too low to demonstrate a therapeutic benefit of extended PLND (11, 23, 24).

Based on these considerations, we used the 2012 Briganti nomogram to select patients for extended PLND and investigated the relationship between NRNs and OS. The results of this study will help to identify PCa patients who may benefit from extended PLND before surgery.

## Patients and Methods

### Patients

PCa patients were identified in the Surveillance Epidemiology and End Results (SEER) database. The SEER database is published by the National Cancer Institute, which collects cancer incidence data from population-based cancer registries covering approximately 28% of the U.S. population (25).

Cases of prostate adenocarcinoma (ICD-0-3 Hist/Behav code = 8,140/3) diagnosed between 2010 and 2015 were included. All of the patients had complete clinicopathological data, namely, clinical T stage, prostate-specific antigen (PSA), Gleason score at biopsy, percentage of positive cores, lymph node status, and survival information. All of the patients underwent radical prostatectomy (surgery code 50) and lymph node dissection. Patients with PSA >50 ng/ml and clinical T4 stage (6th edition of American Joint Committee on Cancer [AJCC] Cancer Stage Manual) were excluded. Furthermore, the PSA value corresponded to the highest lab value documented in the medical record prior to biopsy and treatment.

### Statistical Analyses

Categorical variables are presented as frequencies and proportions. Medians and interquartile ranges (IQRs) are reported for continuously coded variables. Group differences in categorical variables and continuous variables were analyzed using the chi-square test and t-test, respectively.

Extended PLND was defined as NRN >75th percentile (≥12). Propensity score matching (PSM) was used to balance the baseline characteristics with a caliper distance of 0.001 between patients with and without extended PLND based on the age at diagnosis, PSA, Gleason score at biopsy, percentage of positive cores, clinical T stage, and race.

The probability of LNI was calculated using the 2012 Briganti nomogram. First, patients were stratified according to the probability of LNI. Second, univariable and multivariable Cox regression analyses were performed to test the relationship between NRN and survival in patients with values higher and lower than the cutoff value. All of the analyses were repeated using categorical NRN (≥12 vs. <12) and continuously coded NRN. Third, Kaplan–Meier analysis was performed to plot survival curves and determine the 5-year overall survival (OS) and cancer-specific survival (CSS) rates.

SPSS (ver. 22.0; IBM Corp., Armonk, NY, USA) and R software (R software for statistical computing, Vienna, Austria) were used for statistical analyses. P <0.05 was considered to be statistically significant.

## Results

### Baseline Patient Characteristics

A total of 27,690 eligible patients, with a median age of 62 years, were included in the study. Among them, 25,952 had pN0 and 1,738 had pN1 PCa. The clinical and pathological characteristics of participants are summarized in **Table 1**. The median NRN was 6 (IQR: 3–11), and the 75th percentile of NRN was ≥12. The medium numbers of removed nodes were 16 (IQR: 14–21) and 4 (IQR: 2–7) for the NRN ≥12 and NRN <12 groups, respectively. The median follow-up period was 33 months (IQR: 14–52 months). During the follow-up period, 567 (2.0%) of the 27,690 patients in the entire cohort and 261 (2.1%) of 12,400 patients in the PSM cohort died. In addition, 147 (0.53%) of 27,690 and 78 (0.63%) of 12,400 patients died from prostate cancer in the entire cohort and PSM cohort, respectively. When patients were stratified by the 75th percentile of NRN, men with NRN ≥12 had a higher probability of LNI (13.6% vs. 4.2%, respectively; p <0.001), higher PSA value (p <0.001), higher clinical T stage (p <0.001), higher Gleason score (p <0.001), and higher number of positive lymph nodes (p <0.001) compared to those with NRN <12. After PSM of patients with and without extended PLND, 6,200 patients were identified in each group.

**Table 1:** Baseline characteristics for patients underwent radical prostatectomy and pelvic lymph nodes dissection between 2010 and 2015 from the SEER database.

Variables	The entire cohort				Propensity-score matched cohort			
	overall	NRN <12	NRN ≥12	p-value	overall	NRN <12	NRN ≥12	p-value
Number of patients (%)	27,690	21,490	6,200		12,400	6 200	6,200	
Age, yr								
Median (IQR)	62 (57–67)	62 (57–67)	62 (57–67)	0.201	62 (57–67)	62 (57–67)	62 (57–67)	0.378
Race, n(%)								
white	22,343 (80.7)	17,215 (80.1)	5,128 (82.7)	<0.001	10,306 (83.1)	5,178 (83.5)	5,128 (82.7)	0.16
black	3,708 (13.4)	2,982 (13.9)	726 (11.7)		1,469 (11.8)	743 (12.0)	726 (11.7)	
Others	1,639 (5.9)	1,293 (6.0)	346 (5.6)		625 (5.0)	279 (4.5)	346 (5.6)	
PSA at diagnosis, ng/ml								
Median (IQR)	6 (5–10)	6 (5–10)	7 (5–11)	<0.001	7 (5–10)	7 (5–11)	7 (5–10)	0.955
Gleason score at biopsy, n (%)								
<8	21,490 (77.6)	17,202 (79.4)	4,288 (71.1)	<0.001	9,129 (73.6)	4,668 (75.3)	4,461 (72.0)	0.128
≥8	6,200 (22.4)	4,461 (20.6)	1,739 (28.9)		3,271 (26.4)	1,532 (24.7)	1,739 (28.0)	
Clinical T stage, n (%)								
T1	49 (0.2)	42 (0.2)	7 (0.1)	<0.001	17 (0.1)	10 (0.2)	7 (0.1)	0.875
T2	17,366 (62.7)	14,014 (65.2)	3,352 (54.1)		6,709 (54.1)	3,357 (54.1)	3,352 (54.1)	
T3	10,275 (37.1)	7,434 (34.6)	2,841 (45.8)		5,674 (45.8)	2,833 (45.7)	2,841 (45.8)	
Percentage of positive cores, %								
Median (IQR)	42 (25–62)	42 (22–60)	43 (25–67)	0.071	43 (25–67)	43 (25–67)	43 (25–67)	0.886
Number of removed lymph nodes								
Median (IQR)	3 (6–11)	4 (2–7)	16 (14–21)	<0.001	4 (5–11)	5 (2–7)	16 (14–21)	<0.001
Pathological N staging, n (%)								
N0	25,952 (93.7)	20,596 (95.8)	5,356 (86.4)	<0.001	11,203 (90.3)	5,847 (94.3)	5,356 (86.4)	<0.001
N1	1,738 (6.3)	894 (4.2)	844 (13.6)		1,197 (9.7)	353 (5.7)	844 (13.6)	
Number of positive lymph nodes								
0	25,952	20,596 (95.8)	5,356 (86.4)	<0.001	11,203 (90.3)	5,847 (94.3)	5,356 (86.4)	<0.001
1	1,064	642 (3.0)	422 (6.8)		650 (5.2)	228 (3.7)	422 (6.8)	
2	322	1,55 (0.7)	167 (2.7)		240 (1.9)	73 (1.2)	167 (2.7)	
3	142	51 (0.2)	91 (1.5)		121 (1.0)	30 (0.5)	91 (1.5)	
>3	210	46 (0.2)	164 (2.6)		186 (1.5)	22 (0.4)	164 (2.6)	

PSA, Prostate-specific antigen; NRN, Number of removed nodes.

### Cox Regression Analyses

**Figure 1:**
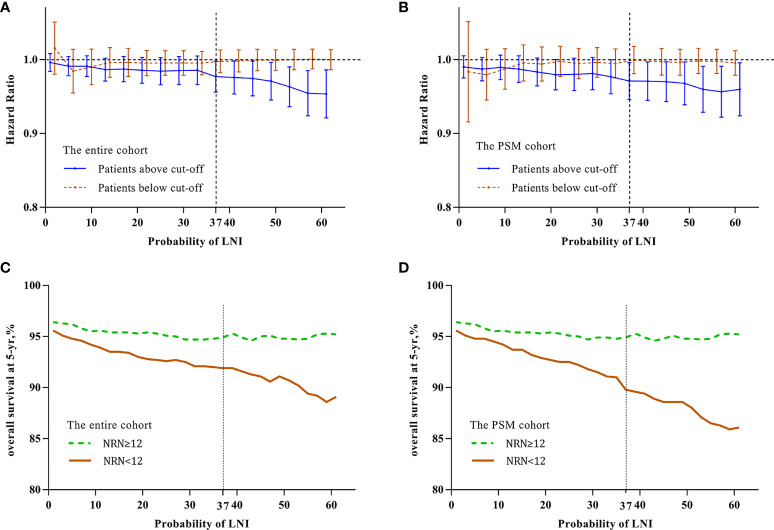
Relationship between hazard ratio (HR) and lymph node invasion (LNI) probability in the entire cohort **(A)** and PSM cohort **(B)**. The HR value was calculated by univariate analysis for continuously coded number of removed nodes (NRN) and overall survival (OS) in prostate cancer (PCa) patients stratified by LNI probability. Brown line indicates patients with LNI probability less than the cutoff value and blue line indicates patients with LNI probability higher than the cutoff value. The results demonstrated that there was a significant difference at a cutoff value above 37 in the entire cohort **(A)** and PSM cohort **(B)**. Kaplan–Meier analysis and log-rank tests were used to estimate 5-y OS in patients with LNI probability higher than the cutoff value in the entire cohort **(C)** and PSM cohort **(D)**. Green line indicates patients with NRN ≥12 and brown line indicates patients with NRN <12. The results demonstrated that patients with NRN ≥12 had significantly higher 5-y OS than those with NRN <12 (p <0.05) when the cutoff value of 37 was used in the entire cohort **(C)** and PSM cohort **(D)**, respectively.

**Figure 2:**
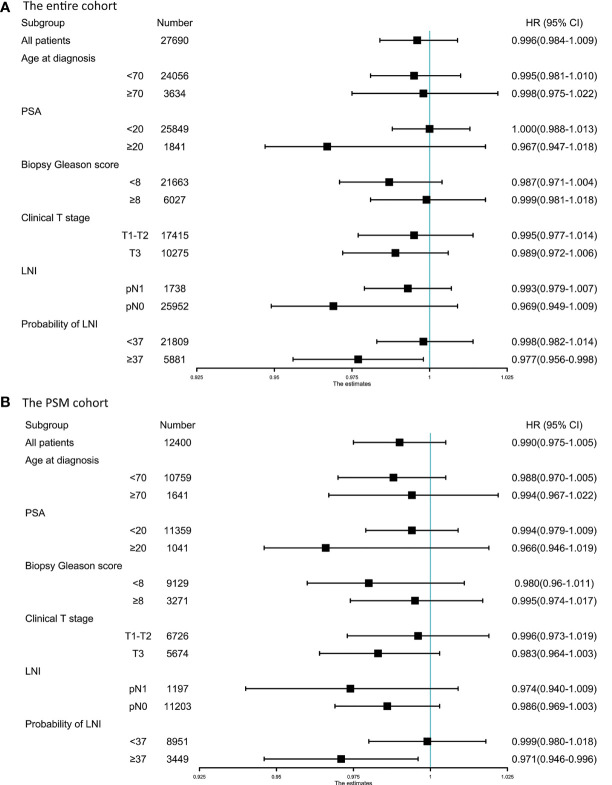
Forest plot of the prognostic effect of pelvic lymph node dissection (PLND) on overall survival of patients with different baseline characteristics in the entire cohort **(A)** and PSM cohort **(B)**.

To identify the optimal cutoff point of probability of LNI to evaluate the benefit of extended PLND, we tested the relationship between continuously coded NRN and survival in patients stratified by different probability of LNI. Univariable Cox regression analysis showed that the hazard ratio (HR) was decreased with an increase in the probability of LNI, and continuously coded NRN was a significant predictor of OS when the cutoff value of probability of LNI ≥37 was used in the entire cohort (all p <0.05; **Figure 1A**) and PSM cohort (all p <0.05; **Figure 1B**). Additionally, to confirm the validity of the cutoff value of 37, Kaplan–Meier analysis and log-rank test were used to estimate the OS at 5 years in patients with NRN ≥12 and NRN <12. We plotted the OS at 5 years against the LNI probability, according to the NRNs. The results showed that patients with NRN ≥12 had a survival benefit when the cutoff was higher than 37 in the entire cohort (**Figure 1C**) and PSM cohort (**Figure 1D**). We further evaluated the relationship between NRN and CSS using univariable analyses, and we found that continuously coded NRN was not an independent predictor of CSS in any LNI risk subgroup (**Supplementary Figures 1A, B** **)**. Although patients with NRN <12 had a faster decline in 5-y CSS rate than those with NRN ≥12, there was no statistical difference between LNI risk subgroups (**Supplementary Figures 1C, D** **)**. Therefore, we selected OS as the end point for further analysis. Subgroup analyses showed that continuously coded NRN was associated with improved OS only in the subgroup with LNI risk ≥37 (**Figure 2A**) and PSM cohort (**Figure 2B**). Based on the above data, LNI risk of 37 was used as the cutoff value to stratify the patients for further analysis.

In the entire cohort, multivariable analyses demonstrated that continuously coded NRN (HR = 0.963, p = 0.002) and categorical NRN (≥12 vs. <12, HR = 0.517, p = 0.001) were independently associated with OS in the population with LNI risk ≥37 after adjustment for all of the covariates, namely, biopsy Gleason score, clinical T stage, PSA, age at diagnosis, percentage of positive cores, and lymph node status (**Table 2**). Similarly, in the PSM cohort, multivariable analyses showed that continuously coded NRN (HR = 0.961, p = 0.004) and categorical NRN (≥12 vs. <12, HR = 0.458, p <0.001) were independently associated with OS in the population with LNI risk ≥37 (**Table 2**). We repeated the analyses using different cutoff values of LNI probability to validate the relationship between NRNs and OS, and we found that NRN was significantly associated with OS in patients with an LNI risk higher than 37, thereby supporting the use of the cutoff value of 37 to select PCa patients for extended PLND (**Table 3**).

**Table 2:** Cox multivariate analyses of prognostic indicators for OS in the entire cohort and PSM cohort.

Variables	The entire cohort		PSM cohort	
	HR (95%CI)	p-value	HR (95%CI)	p-value
Age (continuous)	1.043 (1.020–1.065)	<0.001	1.046 (1.016–1.076)	0.002
Gleason score at biopsy				
≥8 vs. <8	1.654 (1.21–2.261)	0.002	2.020 (1.32–3.091)	0.001
Clinical T stage				
T3 vs. T1–T2	1.327 (0.693–2.543)	0.394	1.145 (0.460–2.85)	0.770
Percentage of positive cores (continuous)	1.011 (1.004–1.018)	0.002	1.012 (1.004–1.021)	0.006
PSA (continuous)	1.003 (0.988–1.018)	0.698	1.001 (0.981–1.020)	0.957
Lymph node invasion				
Yes vs. N0	2.036 (1.464–2.831)	<0.001	1.753 (1.136–2.705)	0.011
NRN (continuous)	0.963 (0.941–0.986)	0.002	0.961 (0.935–0.987)	0.004
NRN				
≥12 vs. <12	0.517 (0.356–0.752)	0.001	0.458 (0.300–0.697)	<0.001

OS, overall survival; PSM, propensity-score matched; NRN， Number of removed nodes; HR, hazard ratio; CI, confidence interval.

**Table 3:** Multivariate analyses of overall survival for patients stratified by the probability of LNI.

The entire cohort					
Probability of LNI, cut-off, %	patients, n	Adjusted HR (95%CI), NRN (continuous)	p-value	Adjusted HR (95%CI), NRN (≥12 vs.<12)	p-value
≥2	26,686	0.985 (0.969–1.005)	0.155	0.772 (0.599–1.078)	0.147
≥9	15,457	0.988 (0.97–1.006)	0.19	0.827 (0.619–1.106)	0.2
≥16	11,148	0.983 (0.963–1.004)	0.111	0.763 (0.547–1.065)	0.112
≥23	8,691	0.973 (0.949–1.002)	0.122	0.699 (0.478–1.022)	0.064
≥30	7,113	0.972 (0.951–1.002)	0.109	0.577 (0.407–0.818)	0.002
≥37	5,881	0.967 (0.944–0.99)	0.005	0.534 (0.365–0.782)	0.001
≥44	4,589	0.962 (0.937–0.987)	0.003	0.529 (0.352–0.794)	0.002
≥51	3,512	0.951 (0.921–0.981)	0.002	0.445 (0.273–0.727)	0.001
≥58	2,549	0.948 (0.915–0.982)	0.003	0.342 (0.19–0.614)	<0.001
**The PSM cohort**					
≥2	12,152	0.985 (0.967–1.004)	0.115	0.723 (0.539–0.092)	0.143
≥9	7,853	0.991 (0.97–1.012)	0.383	0.806 (0.576–1.129)	0.209
≥16	6,010	0.978 (0.955–1.013)	0.077	0.673 (0.463–1.142)	0.134
≥23	4,860	0.967 (0.94–1.023)	0.095	0.637 (0.418–1.109)	0.052
≥30	4,077	0.973 (0.95–1.008)	0.056	0.575 (0.395–0.825)	0.015
≥37	3,449	0.961 (0.935–0.987)	0.004	0.453 (0.298–0.689)	<0.001
≥44	2,783	0.961 (0.934–0.989)	0.006	0.451 (0.289–0.706)	<0.001
≥51	2,173	0.950 (0.917–0.983)	0.004	0.379 (0.222–0.646)	<0.001
≥58	1,626	0.945 (0.908–0.983)	0.005	0.282 (0.150–0.531)	<0.001

Adjusted for Gleason score at biopsy, clinical T stage, PSA, age at diagnosis, percentage of positive cores, and lymph node invasion.

LNI, lymph node invasion; NRN, number of removed nodes; HR, hazard ratio; CI, confidence interval.

### Survival Analyses

**Figure 3:**
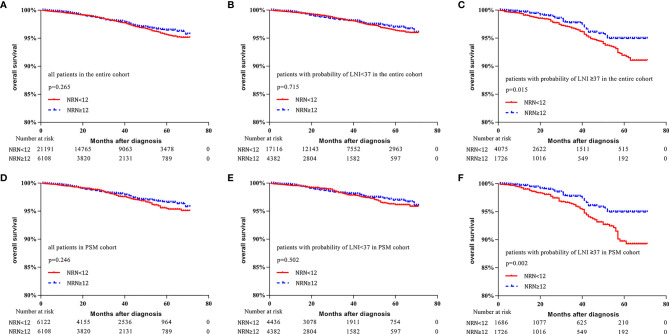
Kaplan–Meier curves of overall survival for patients in the entire cohort **(A)**, patients with lymph node invasion (LNI) probability <37 in the entire cohort **(B)**, patients with LNI probability ≥37 in the entire cohort **(C)**, all patients in the propensity-score matched (PSM) cohort **(D)**, patients with LNI probability <37 in the PSM cohort **(E)**, and patients with probability of LNI ≥37 in the PSM cohort **(F)**. Patients were stratified according to the number of removed nodes (NRN ≥12 vs. NRN <12).

In the entire cohort, no significant difference in survival was found between patients with NRN ≥12 and those with NRN <12 (5-y OS rate: 96.4% vs. 95.6%, respectively, p = 0.265, **Figure 3A**). When the cutoff value of 37 was used to stratify the patients, the survival benefit of extended PLND was not found in patients with LNI risk <37 (5-y OS rate: 97.0% vs. 96.4%, respectively, p = 0.715, **Figure 3B**). However, patients with NRN ≥12 had improved OS compared to those with NRN <12 in the cohort with LNI risk ≥37 (5-y OS rate: 94.9% vs. 91.9%, respectively, p = 0.015, **Figure 3C**).

In the PSM cohort, no significant difference was found in the survival benefit between patients with NRN ≥12 and those with NRN <12 (5-y OS rate: 96.6% vs. 95.5%, respectively, p = 0.246, **Figure 3D**). There was no survival benefit of extended PLND in patients with LNI risk <37 (97.0% vs. 96.2%, respectively, p = 0.502, **Figure 3E**). However, the survival benefit of extended PLND was found in patients with LNI risk ≥37 (94.9% vs. 89.8%, respectively, p = 0.002, **Figure 3F**).

**Figure 4:**
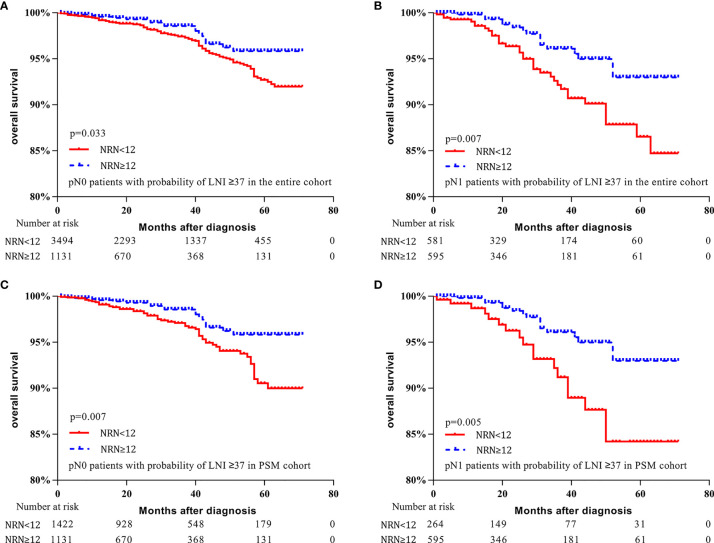
Kaplan–Meier curves of overall survival for pN0 patients with lymph node invasion (LNI) probability ≥37 in the entire cohort **(A)**, pN1 patients with LNI probability ≥37 in the entire cohort **(B)**, pN0 patients with LNI probability ≥37 in the propensity-score matched (PSM) cohort **(C)**, and pN1 patients with LNI probability ≥37 in the PSM cohort **(D)**. Patients were stratified according to the number of removed nodes (NRN ≥12 vs. NRN <12).

Moreover, for patients with LNI risk ≥37 in the entire cohort, OS was improved for pN0 and pN1 patients who had NRN ≥12 (5-y OS rate: 95.8% vs. 92.7%, respectively, p = 0.033, **Figure 4A**; 93.0% vs. 86.5%, respectively, p = 0.007, **Figure 4B**). Similarly, for patients with LNI probability ≥37 in the PSM cohort, OS was improved in both pN0 and pN1 patients who had NRN ≥12 (5-y OS rate: 95.8% vs. 90.5%, respectively, p = 0.007, **Figure 4C**; 93.0% vs. 84.2%, respectively, p = 0.005, **Figure 4D**). These results suggest a survival benefit of extended PLND for patients with an LNI probability ≥37, regardless of the pathological node status, implying that the Briganti nomogram can be used to identify PCa patients who may benefit from extended PLND before surgery.

## Discussion

The therapeutic value of extended PLND for PCa patients is controversial (26). A recent RCT analyzed 1,440 PCa patients and found that extended PLND did not decrease the risk of BCR (12). However, the vast majority of patients in that study were at low or intermediate risk of LNI (27). Another RCT recruited 300 intermediate- and high-risk PCa patients and the researchers reported that extended PLND did not decrease the risks of BCR and CSM. However, the benefit of extended PLND on the BCR was found in patients with ISUP grade groups 3–5 (10). These results suggest that extended PLND may have a potential survival benefit in appropriately selected patients. Previous studies reported that the risk groups based on preoperative PSA level, biopsy Gleason score, and clinical stage did not accurately select PCa patients who may benefit from extended PLND (6, 10, 11). To address this issue, we used the 2012 Briganti nomogram to identify the appropriate PCa patients and evaluated the therapeutic value of extended PLND in a large cohort of PCa patients who underwent RP plus PLND. This study demonstrated that extensive PLND was associated with improved OS in patients with LNI probability ≥37.

Several retrospective studies have explored the relationship between survival of PCa patients and NRNs (15, 28, 29), and they found that a cutoff value of NRN ≥10 or NRN ≥11 was used. Joniau et al. conducted a prospective study and reported that the medium number lymph node counts were 16 (IQR: 10–21) and 6 (IQR: 4–9) for extended PLND and limited PLND, respectively (5). A recent RCT also reported that the median lymph node count was 17 (IQR: 13–24) for extended PLND and 3 (IQR: 2–5) for limited PLND (10). In this study, the 75th percentile of NRN (i.e., 12) was used as the cutoff value. The medium number of removed nodes was 16 (IQR: 14–21) and 4 (IQR: 2–7) for NRN ≥12 and NRN <12 groups, respectively. The analyses were repeated using continuously coded NRN to validate the categorical analyses, which may reduce potential selection bias caused by the use of artificial thresholds.

In the entire cohort, patients with NRN ≥12 harbored more aggressive clinicopathological features, and NRNs were not significantly associated with OS. After using a cutoff LNI value of 37 to stratify the patients, higher NRN was associated with improved OS in patients with LNI risk ≥37. Using PSM to balance the baseline characteristics, the survival benefit of extensive PLND was also found in patients with LNI probability ≥37. These findings suggest that the Briganti nomogram could be used to select patients who may benefit from extended PLND prior to surgery. In addition, the results help to reconcile the previously published data that suggested that extended PLND may have survival benefit for highly selected patients (10, 14, 15). Schiavina et al. analyzed data from 872 patients and found that NRN was not a significant predictor of BCR in the low-risk group, while patients with NRN ≥10 had improved BCR survival in the intermediate- and high-risk groups (15). In another report, Moschini et al. examined data from 1,586 pT3–T4 PCa patients treated with RP plus extended PLND, and they found that a higher NRN was associated with a lower CSM rate (14).

Compared to previous studies that focused on pN0 or pN1 patients, our study included both pN0 and pN1 patients, and it may be able to minimize the effect of the Will Rogers phenomenon (30). Patients with more extensive PLND were more likely to be accurately staged, whereas patients with limited PLND were less likely to be accurately staged, harbor occult metastases, and have worse prognoses. Our study showed a survival benefit of extended PLND for patients with LNI probability ≥37, regardless of the pathological node status, which supports the use of the Briganti nomogram to identify PCa patients who may benefit from extended PLND before surgery.

For pN0 patients, extensive PLND significantly improved the OS in patients with LNI probability ≥37. This finding is consistent with those from earlier studies (15, 28, 29, 31, 32). A potential explanation for these results is that extended PLND may eliminate occult metastases. Pagliarulo et al. reported that occult metastases were found in 13.3% of pT3 patients who were staged as node negative based on routine histologic evaluation (33). In our study, 92.0% of pN0 patients with LNI probability ≥37 had T3 stage, and these patients had a higher likelihood of occult metastases. For pN1 patients, a survival benefit of extended PLND was observed in patients with LNI probability ≥37. The results were in accord with those of previous studies (13, 31, 34). Abdollah et al. found that a higher NRN was associated with a lower CSM rate in patients with pN1 disease (13). Moreover, in a prospective study, Joniau et al. showed that extended PLND removed all of the positive lymph nodes in 76% (26/34) of patients, which was higher than that with limited PLND (29% of patients) (5). These results suggest that patients with LNI may benefit from extended PLND by completely removing metastases at the time of surgery.

Although there was a trend toward CSS benefit with extended PLND in patients with high LNI risk, it was not statistically significant. With the increase in LNI risk, patients with NRN <12 had more rapid decline in 5-y CSS rate compared to those with NRN ≥12. For patients with LNI risk ≥37 in the PSM cohort, the 5-y CSS rates were 94.4 and 96.4% for patients with NRN <12 and NRN ≥12, respectively (p = 0.174). When the cutoff LNI risk value of 57 was used in the PSM cohort, the 5-y CSS rates were 92.6 and 96.3% for patients with NRN <12 and NRN ≥12, respectively (p = 0.110). The lack of effect on CSS rate may be due to the short follow-up duration. Moschini et al. found that a higher NRN was associated with a lower CSM rate in locally advanced PCa with a median follow-up duration of 72 months (14). Additionally, patients with NRN ≥12 had similar 5-y OS and 5-y CSS rates, while patients with NRN <12 had a more rapid decline in 5-y OS rate compared to CSS rate (**Figure 1D** and **Supplementary Figure 1D**), implying that additional treatment to prevent PCa progression in patients with NRN <12 may lead to more deaths from other causes. PCa patients treated with ADT had increased risks of thrombosis, myocardial infarction, severe arrhythmia, and sudden cardiac death (35–38). The possibility of selection bias due to the extraction of data from the SEER database should also be considered. The impact of extended PLND on CSS should be further investigated in prospective studies with long-term follow-up.

Our study results demonstrate OS benefit with extensive PLND in patients with LNI risk ≥37. These results are important to select patients who may benefit from extended PLND. Even so, the results were limited because patients were stratified based on clinical variables. A novel nomogram, namely, multiparametric magnetic resonance imaging to identify patients for extended PLND and a model, had high accuracy for predicting LNI (17). Moreover, a recent research reported a Node Reporting and Data System 1.0 (Node-RADS) for standardized reporting of possible distant lymph node involvement on CT and MR imaging (19). The novel system will lead to an increase in the consensus over radiological assessment of the lymph nodes in PCa patients. With the development of imaging techniques, novel molecular classification, and artificial intelligence, the use of clinical variables alone for risk stratification will become obsolete. Moreover, psychosocial factors are important to consider when selecting patients for extended PLND. Sociodemographic parameters, such as level of education, age, family support, and employment status, can influence the psychology of the primary treatments for PCa patients, thereby affecting the treatment results and likelihood of necessary follow up (39). The opinions of patients should be considered when making treatment decisions.

Several unavoidable limitations existed in our study. First, there was no information in the SEER database about preoperative or postoperative treatment, including ADT, chemotherapy time and dosage, and site-specific radiation therapy. Therefore, the effects of these treatments on the survival of PCa patient needs further investigation. Second, extended PLND was defined using the NRNs rather than an anatomical template, owing to the lack of information on anatomical template in the SEER database. Although several studies indicated that NRNs may serve as a surrogate for the anatomical template (29, 40, 41), anatomical templates are considered as a superior method to define extended PLND because NRNs vary between studies (5, 10, 12). Third, this was a retrospective, observational analysis of PCa patients with a median follow-up duration of 33 months. The study results need to be further validated in prospective trials with long-term follow up. Fourth, patients diagnosed between 2010 and 2015 were included because the percentage of biopsy cores were available for the 2012 Briganti nomogram for these patients. Finally, our results suggest a survival benefit of extended PLND for PCa patients with LNI probability ≥37, but does not imply that patients with LNI probability <37 can be omitted from consideration for extended PLND. The therapeutic impact of extended PLND in patients with LNI probability of 5–37 needs further investigation.

### Conclusion

Extensive PLND may be associated with improved survival in PCa patients with LNI probability ≥37. The results support the use of extended PLND for highly selected PCa patients. The Briganti nomogram can be used to identify patients who may benefit from extended PLND before surgery. Prospective trials with long-term follow up are needed to further evaluate the role of extended PLND in PCa patients with a high LNI risk.

## Data Availability Statement

The original contributions presented in the study are included in the article/**Supplementary Material**. Further inquiries can be directed to the corresponding authors.

## Author Contributions

Conception and design: BY, XY, and GY. Acquisition of data: GY, JX, and YG. Analysis and interpretation of data: GY, JY, RW, and CG. Writing, review, and/or revision of the manuscript: GY, BY, and JX. Administrative, technical, or material support: XY and BP. Study supervision: BY and XY. All authors contributed to the article and approved the submitted version.

## Funding

This work was partly supported by the National Natural Science Foundation of China (grant No. 31570993) and the Shanghai Pujiang Program (grant No. 15PJ1407000).

## Conflict of Interest

The authors declare that the research was conducted in the absence of any commercial or financial relationships that could be construed as a potential conflict of interest.

## Publisher’s Note

All claims expressed in this article are solely those of the authors and do not necessarily represent those of their affiliated organizations, or those of the publisher, the editors and the reviewers. Any product that may be evaluated in this article, or claim that may be made by its manufacturer, is not guaranteed or endorsed by the publisher.

## Supplementary Material

Supplementary Figure 1 The relationship between hazard ratio (HR) and lymph node invasion (LNI) probability in the entire cohort **(A)** and the PSM cohort **(B)**. The HR value was calculated by univariate analysis for continuously coded number of removed nodes (NRN) and cancer-specific survival (CSS) in prostate cancer (PCa) patients stratified by LNI probability. Brown line indicates patients with LNI probability less than the cutoff value and blue line indicates patients with LNI probability higher than the cutoff value. The results demonstrated that continuously coded NRN was not an independent predictor of CSS in any LNI risk subgroup on univariable analyses in the entire cohort (A) and PSM cohort **(B)**. Kaplan-Meier analysis and log-rank tests were used to estimate 5-y CSS in patients with LNI probability less than the cutoff value in the entire cohort **(C)** and PSM cohort **(D)**. Green line indicates patients with NRN ≥ 12 and brown line indicates patients with NRN < 12. The results demonstrated that patients with NRN < 12 had more rapid decline in 5-y CSS rate than those with NRN ≥ 12, but there was no statistical difference in any LNI risk subgroups in the entire cohort **(C)** and PSM cohort **(D)**.
