# Supplementary figures and images for: Identifying the Candidates Who Will Benefit From Extended Pelvic Lymph Node Dissection at Radical Prostatectomy Among Patients With Prostate Cancer
Source: Front Oncol. 2022 Jan 26;11:790183. doi: 10.3389/fonc.2021.790183 (PMC8826072; doi:10.3389/fonc.2021.790183)

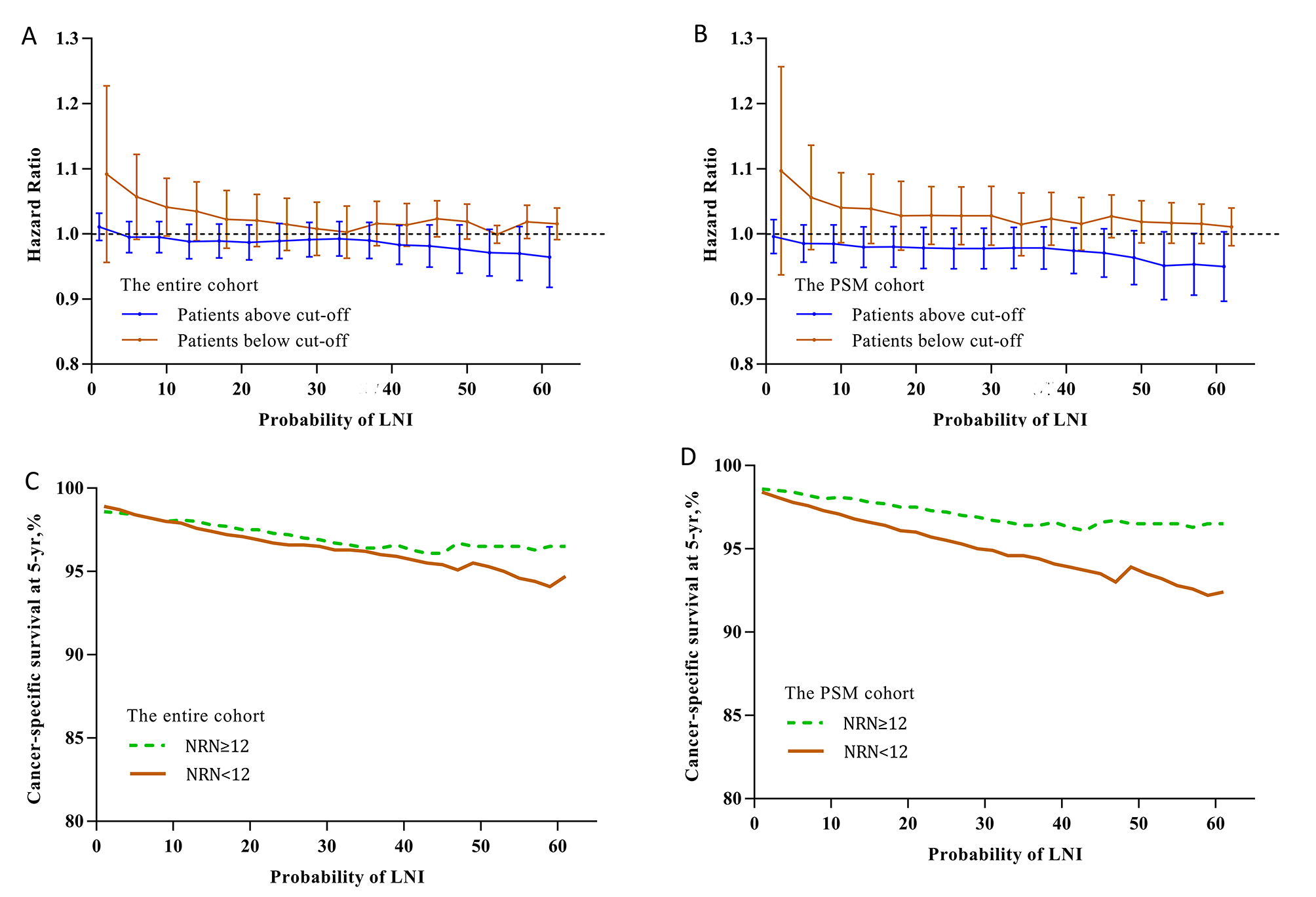

Supplement: Supplementary Figure 1 — The relationship between hazard ratio (HR) and lymph node invasion (LNI) probability in the entire cohort (A) and the PSM cohort (B). The HR value was calculated by univariate analysis for continuously coded number of removed nodes (NRN) and cancer-specific survival (CSS) in prostate cancer (PCa) patients stratified by LNI probability. Brown line indicates patients with LNI probability less than the cutoff value and blue line indicates patients with LNI probability higher than the cutoff value. The results demonstrated that continuously coded NRN was not an independent predictor of CSS in any LNI risk subgroup on univariable analyses in the entire cohort (A) and PSM cohort (B). Kaplan-Meier analysis and log-rank tests were used to estimate 5-y CSS in patients with LNI probability less than the cutoff value in the entire cohort (C) and PSM cohort (D). Green line indicates patients with NRN ≥ 12 and brown line indicates patients with NRN < 12. The results demonstrated that patients with NRN < 12 had more rapid decline in 5-y CSS rate than those with NRN ≥ 12, but there was no statistical difference in any LNI risk subgroups in the entire cohort (C) and PSM cohort (D). [file Image_1.tif]
